# A review of pretreatment methods and detection techniques for nonylphenol

**DOI:** 10.3389/fpubh.2026.1821020

**Published:** 2026-05-29

**Authors:** Hao Yao, Jie Yu, Jie Xu

**Affiliations:** School of Public Health, Zunyi Medical University, Zunyi, Guizhou, China

**Keywords:** detection techniques, ecological risk assessment, endocrine disruptor, nonylphenol, pretreatment methods

## Abstract

Nonylphenol (NP) is a ubiquitous endocrine-disrupting chemical with confirmed adverse effects on multiple human physiological systems, posing urgent demands for its accurate detection in environmental and biological matrices. This review aims to systematically summarize the pretreatment methods and mainstream detection techniques for NP, with a critical synthesis of their analytical performance, applicable scenarios, advantages, and limitations. Core comparative findings are clarified: chromatography-mass spectrometry technologies (LC-MS/MS, GC-MS) are the gold standard for trace NP detection in complex matrices, with differentiated pretreatment requirements; HPLC has wide applicability but limited sensitivity for ultra-trace analysis; immunological assays are suitable for rapid on-site screening but with high detection limits. This work provides standardized guidance for NP detection method selection, directly supporting environmental monitoring and health risk assessment of NP.

## 1 Introduction

**Figure 1:**
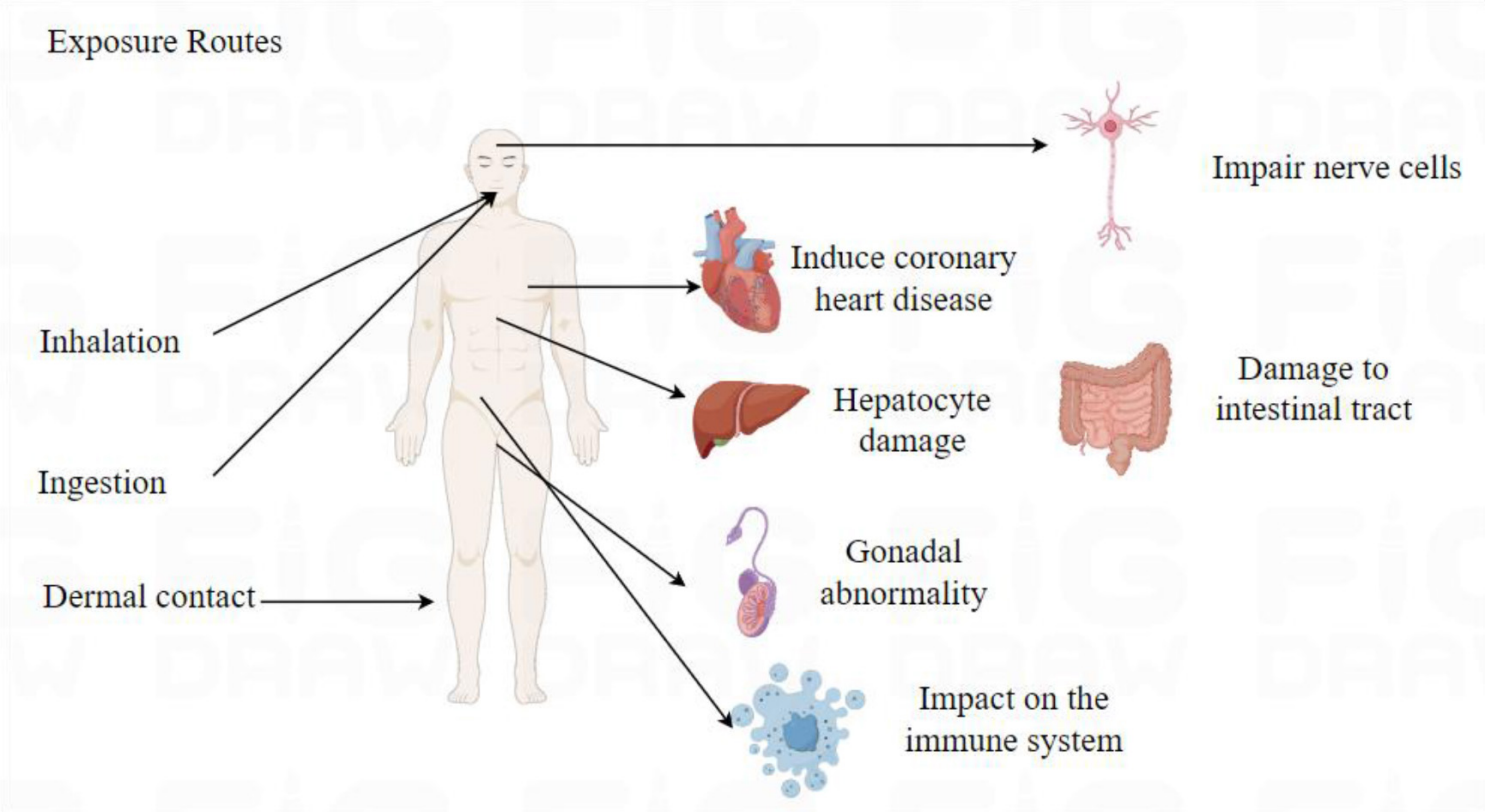
Pathways to human body and adverse health effects of nonylphenol.

Nonylphenol (NP) is a globally concerned environmental endocrine-disrupting chemical (EDC), which has been widely investigated by domestic and international research groups (1). It is extensively used as a key raw material in the industrial production of nonionic surfactants, antioxidants, textile printing and dyeing auxiliaries, and other fine chemical products. A large number of studies have confirmed that NP can interfere with the endocrine function of humans and a wide range of animal species, exerting adverse effects on the reproductive, immune, nervous, and cardiovascular systems, with proven carcinogenic, teratogenic, and estrogenic effects (1). NP in the environment mainly originates from industrial wastewater discharge, domestic waste leaching, and the degradation of NP-containing products, and is widely detected in various environmental matrices including surface water, groundwater, soil, and air, as well as in food matrices such as raw milk, aquatic products, and plastic-packaged foods. NP can enter the human body through multiple exposure pathways including respiratory inhalation, digestive tract intake, and dermal contact. Notably, NP has strong bioaccumulation and biomagnification effects in aquatic ecosystems: it can be enriched in aquatic organisms at multiple trophic levels, and further enter the human body through the food chain via the consumption of aquatic products, posing a continuous and cumulative health risk to humans (2) (Figure 1). Accurate, sensitive, and matrix-adapted detection of NP is therefore the core prerequisite for its environmental exposure monitoring, pollution source traceability, and human health risk assessment.

At present, the mainstream detection technologies for NP worldwide can be divided into two categories: laboratory-based precision instrumental analysis methods and on-site rapid screening methods. The former mainly includes high-performance liquid chromatography (HPLC) (3), liquid chromatography-tandem mass spectrometry (LC-MS/MS) (4), gas chromatography-tandem mass spectrometry (GC-MS/MS) (5), capillary electrophoresis (CE) (6), micellar electrokinetic chromatography (MEKC), spectroscopic methods (7), and electrochemical detection (8); the latter mainly includes immunological detection technology, microarray technology, and yeast two-hybrid technology. Conventional chromatographic and mass spectrometric methods have the advantages of high accuracy, low detection limits, and good stability, and are the gold standard methods for NP quantitative detection in most national and international standards. However, these methods usually require complex sample pretreatment, long detection cycles (several hours to more than 10 h), expensive instruments, and professional operators, which cannot meet the demand for high-throughput and on-site rapid screening of large batches of samples. In contrast, rapid detection technologies represented by immunological methods can complete sample detection within a few minutes, with the advantages of simple operation, low cost, high specificity, and on-site detection potential, but they generally have higher detection limits and lower sensitivity than precision instrumental methods, and are difficult to meet the requirements for ultra-trace NP quantification in complex matrices.

Although multiple reviews on NP detection have been published in recent years, most of the existing literature focuses on a single type of detection technology (e.g., only mass spectrometry methods or only rapid detection methods) or is limited to NP detection in a specific matrix (e.g., only water samples or only food samples). There is still a lack of a systematic and comprehensive review that simultaneously integrates sample pretreatment protocols, mainstream precision instrumental detection methods, and on-site rapid screening technologies, and conducts a full-dimensional comparative analysis of their applicable matrices, analytical performance, inherent advantages and limitations, and detection constraints. Meanwhile, with the continuous tightening of NP limit standards in environmental and food matrices worldwide, there is an urgent need for a scenario-based methodological guidance framework to help researchers and testing institutions select the appropriate NP detection method for different sample types and detection purposes, so as to improve the accuracy and reliability of detection results.

To fill the above research gap, this paper systematically reviews the research progress of NP detection technologies at home and abroad in recent years. We focus on the core dimensions including detection principle, applicable sample matrices, advantages and disadvantages, and detection limitations of each method, and further provide a clear guidance for the selection of NP detection methods under different application scenarios. This review aims to provide a systematic reference for the standardized detection of NP in environmental and food matrices, and to support the development of NP pollution control and health risk assessment research.

## 2 Nonylphenol pretreatments before extraction

Due to the trace concentrations of nonylphenol (NP) in environmental and biological matrices, efficient pretreatment is essential to enrich and concentrate the analyte prior to detection. The selection of a pretreatment method is primarily governed by the sample matrix (solid, liquid, or gas) and the complexity of the interfering components. A comparative summary of these methods, including their application across different matrices and their respective performance metrics, is provided in Table 1.

**Table 1:** Nonylphenol pretreatment methods, applicable media, advantages, and disadvantages.

Pretreatment methods	Solid phase extraction	Liquid-liquid extraction	Soxhlet extraction	Solid phase microextraction
Advantages	Less consumption, time saving, automation	Room temperature operation, no phase change, high separation coefficient	High selectivity, low energy consumption, simple operation	Simple operation, no solvent, easy elution
Disadvantages	The extraction amount is small, easy to clog, and needs further purification	Easy to form emulsion, need phase separation	The extraction time is long, the amount of solvent is large, and special instruments are needed	The extraction phase is easy to wear
Applicable medium	Solid and liquid	Liquid	Solid	Solid, liquid, and gas

### 2.1 Solid-phase extraction (SPE)

Solid-phase extraction (SPE) is a cornerstone technique for NP analysis, favored for its high enrichment factors, low solvent consumption, and potential for automation. Its versatility allows for the processing of both liquid and solid samples (following initial extraction). Recent advancements have focused on the development of novel adsorbents to improve selectivity and recovery. For instance, Liu et al. (9) utilized amino-functionalized covalent organic framework nanofibers to extract NP from urine, achieving a low detection limit of 0.02 ng/mL and satisfactory recoveries. In complex food matrices, such as hot-pot bases and spicy condiments, composite fillers like multi-walled carbon nanotubes/PSA have been successfully employed to mitigate matrix interference (10). Furthermore, the integration of nanotechnology, such as the use of magnetic covalent organic framework nanocomposites, has streamlined the process by facilitating rapid phase separation in environmental water samples (11). While SPE offers superior sensitivity and reproducibility, it is constrained by the potential for column clogging in suspended-solid-rich samples and the relatively high cost of specialized cartridges.

### 2.2 Liquid-liquid extraction (LLE)

Liquid-liquid extraction (LLE) remains a standard approach for aqueous samples, offering a straightforward mechanism without phase changes. However, traditional LLE is often hindered by the formation of emulsions and high organic solvent requirements. To address these limitations, microextraction variants have been developed. Wang et al. (12) demonstrated the efficacy of hydrophobic deep eutectic solvents for extracting NP from vegetable oils, significantly simplifying the cleanup process for lipid-rich samples compared to traditional methods. Similarly, liquid-phase microextraction techniques, such as those utilizing glass surface etching or continuous flow extraction systems, have been applied to bottled and environmental water (13, 14). These methods maintain high recovery rates (typically >90%) while drastically reducing solvent waste, making them suitable for routine monitoring of liquid matrices.

### 2.3 Soxhlet extraction

Soxhlet extraction is the conventional choice for solid matrices, particularly those with high lipid content or complex structures like river sediments. Its primary advantage lies in its high selectivity and the ability to handle finely ground solid samples that might otherwise agglomerate in other systems (15, 16). Santos et al. (17) utilized this method to achieve stable recoveries (82–106%) from estuarine sediment samples. Despite its reliability and simple equipment requirements, the technique is increasingly being scrutinized in modern reviews due to its long extraction times and excessive solvent consumption (18, 19), which may not align with “green chemistry” objectives.

### 2.4 Solid-phase microextraction (SPME)

Solid-phase microextraction (SPME) represents a solvent-free alternative that integrates sampling, extraction, and concentration into a single step. It is uniquely applicable across gas, liquid, and solid (headspace) phases. The technique's performance is highly dependent on the coating properties of the fibers. Research into crosslinked polymer ionic liquid-based coatings has shown that SPME can achieve high selectivity for NP in water samples, with stable recoveries of 82% to 104% under optimized conditions (20). Gómez-Ramos et al. (21) further validated the practical utility of automated headspace SPME-GC-MS/MS by detecting ultra-trace NP in surface and drinking water samples across Spain. While SPME offers rapid analysis and easy operation, the fragility and limited lifespan of the extraction fibers remain the primary technical drawbacks.

## 3 Detection method of nonylphenol

### 3.1 High-performance liquid chromatography (HPLC)

HPLC is a common technique for detecting nonylphenol and can be used to analyze contaminants in environmental samples. HPLC is also widely used in food analysis, such as detecting milk powder (22) and pesticide residues (23). The principle of HPLC is mainly based on the difference in partition coefficients of the components of the sample between the stationary phase and the mobile phase. The sample solution is passed under high pressure through a column filled with a highly fine-grained, high-performance stationary phase. Due to the difference in size and strength of the forces between the different components and the stationary phase, their retention times in the stationary phase will also be different, thus achieving separation of the components. HPLC can only measure the concentration of samples whose concentration is above the limit of detection, i.e., the limit of quantification, which is usually the minimum sample concentration that an HPLC system can accurately detect. HPLC can detect nonylphenol in water, soil, and serum samples, and it is commonly used to detect nonylphenol in water. Ma et al. (24) prepared a magnetic molecularly imprinted polymer based on metal-organic frameworks, and used it as an extractant to extract nonylphenol from lake water, with detection by HPLC. The results showed detection limits of 0.08–0.25 μg/L, recoveries of 90.2%−103.1%, and relative standard deviations of <2.3%. Li et al. (25) chose a nonionic surfactant synergistic cloud point extraction system as the pretreatment method, and the detection limit of nonylphenol in water was 0.12–0.31 μg/L by HPLC. Kim et al. (26) first proposed a novel deep eutectic solvent-based in-tube dispersive liquid-liquid microextraction (DES-IT-DLLME) technique, and used it as a simple, rapid, and environmentally friendly pretreatment method for HPLC determination of nonylphenol in aqueous samples, with a detection limit of 0.52 μg/L and recoveries ranging from 93.4% to 106.8%. The sample extraction was completed in less than 3 min, and only green deep eutectic solvents were used, demonstrating the advantages of low cost, simplicity, rapidity and environmental friendliness.

#### 3.1.2 HPLC advantages and disadvantages

Stationary phase particles (below 10 μm) used in HPLC columns are tiny, so it has a high separation efficiency. In addition to the high separation efficiency, HPLC is characterized by high speed, high sensitivity, and a high degree of automation. Moreover, the HPLC method can analyze compounds with high boiling points, poor thermal stability, and large molar masses. However, the method has a high detection cost and an extended analysis time.

### 3.2 Liquid chromatography-tandem mass spectrometry (LC-MS/MS)

Liquid chromatography-tandem mass spectrometry (LC-MS/MS) is an effective means of separating and analyzing complex mixtures by combining the ability of liquid chromatography to efficiently separate thermally unstable and high-boiling-point compounds with the strong component identification ability of mass spectrometry. LC-MS/MS principles mainly include liquid chromatography separation and mass spectrometry detection and identification. The principle of LC-MS/MS separation is that liquid chromatography separates molecules based on their partition and affinity properties in the liquid phase. The sample is dissolved in the mobile phase and separated into different components by the stationary phase (column). These components pass through the column at different rates due to different interactions (e.g., polarity, partition coefficient, etc.), separating the components. The principle of mass spectrometry detection and identification is that the separated sample components sequentially enter the mass spectrometry detector, where the sample molecules are converted to ions by ionization in the mass spectrometer. There are various ionization methods, one of which is electrospray ionization (ESI), which is one of the most commonly used and which can use the large mass of organic molecules to generate multi-charged ions. The ionized ions move in an electromagnetic field and are separated according to their mass-to-charge ratio (m/z). Different ions behave differently in the electromagnetic field so that a mass analyzer can separate the ions according to different mass-to-charge ratios. The separated ion beam enters a detector, which records and measures the intensity of each ion peak to produce a mass spectrogram. The mass spectrogram shows the mass-to-charge ratio distribution and the relative content of each component in the sample, using the mass-to-charge ratio of the ions as the horizontal coordinate and the intensity of the ion signal (i.e., ion abundance) as the vertical coordinate.

#### 3.2.1 Applicable media for LC-MS/MS

LC-MS/MS can be used to determine nonylphenol in serum, food, and air samples and is commonly used for detecting nonylphenol in solid and liquid samples. Zhang et al. (27) used LC-MS/MS for the determination of nonylphenol residues in infant milk powder, with a detection limit of 2 μg/kg, recoveries of 87.3%−105.9%, and relative standard deviations of 2.62%−8.74%. The method is simple and accurate and can be used for the determination of nonylphenol residues in infant milk powder. Furthermore, the same research group extracted nonylphenol from various food matrices with acetonitrile, removed protein, separated on a Hypersil GOLD C18 chromatographic column with a methanol-water mobile phase, and analyzed NP by LC-MS/MS. The limits of detection were 0.030 μg/kg and the limits of quantification were 0.080 μg/kg for packaged drinking water, with relative standard deviations of 2.7%−5.3%. The limits of detection and limits of quantification of other foodstuffs were 0.80 μg/kg and 1.50 μg/kg, with relative standard deviations of 2.6%−9.1%. The experimental results demonstrated that the method has good sensitivity and is suitable for the determination of nonylphenol in various foodstuffs. Notably, this technique has also been extended to the detection of NP in complex industrial matrices, with Wang et al. (28) establishing an LC-MS method for the quantitative determination of NP in crude oils and petroleum products, providing technical support for NP pollution control in the petroleum refining industry and sustainable production.

#### 3.2.2 Advantages and disadvantages of LC-MS/MS

LC-MS/MS combines both liquid chromatography and mass spectrometry, and can improve the lower detection limit over liquid chromatography alone, solving the problem of accelerated characterization by mass spectrometry after separation by liquid chromatography. This sensitive method has a low detection limit and can be used to detect substances such as nonylphenol, which are present at ultra-trace levels in the environment. In addition, using liquid chromatography-tandem mass spectrometry can improve the precision of detection and the accuracy of the results and shorten the sample processing time. However, solid samples need to be dissolved in a suitable solvent to be detected by LC-MS/MS, and this technique is expensive.

### 3.3 Gas chromatography-mass spectrometry (GC-MS)

Gas Chromatography-Mass Spectrometry (GC-MS) is a technology that combines two techniques: gas chromatography and mass spectrometry. This technique uses gas chromatography as a means of separation and mass spectrometry as a means of identification, and the combination of the two constitutes a gas chromatography-mass spectrometry coupling technique. The principle of GC-MS can be divided into two main steps: gas chromatographic separation and mass spectrometric identification. First, the sample is driven by a carrier gas into a chromatographic column. The column is filled with a specific solid or liquid packing (a stationary phase) with different chemical properties. Different compounds in the sample have different interactions (e.g., adsorption or partition coefficients) with the stationary phase, causing them to move through the column at different rates. As a result, the compounds are gradually separated into separate components in the column. The least adsorbent components leave the column first and enter the detector, while the most adsorbent leave last. The components leave the column in the order of their retention time in the column. The separated components flow into the detector with the carrier gas, and the detector converts the change in concentration or mass of the compound into an electrical signal, which is recorded to form a chromatographic effluent curve. Each peak on the chromatographic efflux curve represents a mixture of one or more compounds, which can be analyzed qualitatively based on the retention time of the peaks. In contrast, the peak area or height is used for quantitative analysis. After entering the mass spectrometer, the components flowing out of the column are first bombarded by an ionization source (e.g., electron bombardment source, chemical ionization source, etc.) to produce charged ions. The ions are separated according to their mass-to-charge ratio under the action of an electric or magnetic field. Ions with different mass-to-charge ratios arrive at the detector at different times. The detector records the mass-to-charge ratio of each ion and its relative intensity to produce a mass spectrogram. The mass spectrum reflects the molecular weight and structural information of each component in the sample.

#### 3.3.1 Applicable media for GC-MS

GC-MS is commonly used to determine nonylphenol in food, air, and water matrices. Wu et al. (29) applied a silica/N-(n-propyl) ethylenediamine hybrid solid-phase extraction cartridge to the extraction of nonylphenol from vegetable oils, with detection by GC-MS, achieving limits of detection and quantification of 0.65 μg/kg and 2.0 μg/kg, respectively. In spiking experiments of vegetable oils, the recoveries of nonylphenol ranged from 72.4%−91.6%. Gómez-Ramos et al. (21) used solid-phase microextraction coupled with GC-MS/MS to determine nonylphenol in water, with a detection limit as low as 0.005 μg/L. de Souza et al. (30) used GC-MS/MS for the determination of nonylphenol in cereal grains, with recoveries in the range of 72%−115%, a relative standard deviation of <12%, and limits of detection in the range of 0.02–3.7 ng/g. The results showed that the method has excellent separability, high selectivity, and a low detection limit. For the complex sewage and sludge matrices from wastewater treatment plants, Liu et al. (31) developed a GC-FID-MS method combined with deans switch technology, which realized the efficient separation and accurate detection of NP isomers in sewage and sludge, further expanding the application of GC-MS technology in the trace analysis of NP in complex environmental solid-liquid mixed matrices.

#### 3.3.2 GC-MS advantages and disadvantages

GC-MS has high accuracy and reliability of results, excellent separation efficiency, and strong qualitative ability for target compounds. However, the technique requires derivatization pretreatment for thermally unstable and highly polar phenolic compounds, and the operation process is relatively complex, which limits its high-throughput application to a certain extent.

### 3.4 Immunobiotechnology

Immunobiotechnology is an emerging technique that uses immunological techniques to detect environmental nonylphenol. The principle of immunobiotechnology is mainly based on the detection of samples based on the specificity between antigens and antibodies, and most of the existing studies have used enzyme-linked immunosorbent assay (ELISA) techniques to detect nonylphenol.

#### 3.4.1 Applicable media for immunobiotechnology

At present, the detection of nonylphenol using immunobiological techniques at domestic and foreign labs is mainly carried out for water bodies, with increasing research expanding to food and biological matrices. Jia et al. (32) used an indirect competitive enzyme immunoassay technique for the determination of nonylphenol in water, with a detection limit of 24.78 μg/L and a quantitative range of 36.51–363.43 μg/L. The coefficients of variation for all samples ranged from 2% to 14%, and the recoveries ranged from 85% to 116%. The results showed that the technique has good precision and accuracy, indicating that it can be used for the detection of nonylphenol in real water samples. Jia et al. (33) used an indirect competitive enzyme immunoassay technique based on the suspension array technique for the determination of nonylphenol in wastewater, with limits of detection ranging from 0.0010 ng/mL to 0.0070 ng/mL, which is more specific and sensitive than the traditional ELISA technique. In addition, the team assayed spiked milk and tap water samples with recoveries in the range of 85%−110%, which showed good repeatability and reproducibility, illustrating the great potential of this technique for the detection of nonylphenol in real samples. Park et al. (34) further developed a monoclonal antibody-based indirect competitive ELISA, which achieved ultra-sensitive detection of NP in environmental water and vegetable samples, with a detection limit of 0.12 μg/L, greatly improving the sensitivity of immunological detection for NP in complex matrices.

#### 3.4.2 Advantages and disadvantages of immunobiotechnology

Compared with conventional instrumental methods, detecting nonylphenol by immunobiotechnology can save costs and shorten the detection time. In addition, immunobiotechnology also has a high specificity. However, the detection limit for immunobiotechnology is generally higher than that of instrumental detection, and the sensitivity is not as high as that of conventional instrumental detection, and there is a risk of cross-reaction with structural analogs.

### 3.5 Critical comparative analysis of mainstream chromatographic-mass spectrometric techniques

While the preceding sections have individually described the principles and applications of HPLC, LC-MS/MS, and GC-MS for NP detection, a systematic critical comparison across key performance metrics is essential to guide rational method selection. This section quantitatively and qualitatively compares these three gold-standard techniques in terms of sensitivity/LOD, analytical cost, and practical applicability, highlighting their complementary strengths and inherent limitations.

#### 3.5.1 Sensitivity and limit of detection

Sensitivity and LOD are the most critical parameters for trace NP detection, especially in complex environmental and biological matrices where NP concentrations often reach nanogram or even picogram levels. LC-MS/MS exhibits the highest overall sensitivity among the three techniques, with reported LODs as low as 0.030 μg/kg in packaged drinking water and 0.80 μg/kg in general food matrices (Section 3.2.1). Its multiple reaction monitoring (MRM) mode effectively eliminates matrix interference, enabling reliable quantification of ultra-trace NP in serum, food, and air samples without extensive purification. GC-MS/MS follows closely, achieving LODs down to 0.005 μg/L in water samples when coupled with SPME pretreatment (Section 3.3.1) and 0.02 ng/g in cereal grains. However, its sensitivity is highly dependent on derivatization efficiency for polar phenolic compounds like NP; incomplete derivatization can significantly increase LODs and reduce quantitative accuracy. HPLC has the lowest sensitivity of the three, with typical LODs ranging from 0.08 μg/L to 0.52 μg/L in water samples (Section 3.1). While fluorescence detection (FLD) can improve sensitivity compared to UV detection, it still cannot match the performance of mass spectrometry-based methods for ultra-trace analysis.

#### 3.5.2 Analytical cost and operational requirements

Cost and operational complexity are major factors influencing the adoption of detection methods in routine laboratories. LC-MS/MS has the highest capital and operational costs. The instrument itself is significantly more expensive than HPLC or GC-MS, and it requires highly trained operators for method development, maintenance, and data interpretation. Additionally, LC-MS/MS consumes large quantities of high-purity solvents and gases, increasing long-term running costs. GC-MS has moderate capital costs but high operational complexity due to the need for derivatization pretreatment for NP analysis. Derivatization steps add significant time to the analytical workflow and introduce potential sources of error, increasing labor costs and reducing sample throughput. HPLC has the lowest capital and operational costs. The instrument is widely available in most analytical laboratories, and routine operation and maintenance are relatively simple. HPLC methods also require less specialized training, making them suitable for high-throughput routine monitoring when ultra-trace sensitivity is not required.

#### 3.5.3 Applicability and matrix compatibility

The choice of detection method is ultimately determined by the sample matrix and the specific analytical objectives. LC-MS/MS is the most versatile technique, applicable to a wide range of matrices including serum, food, water, soil, and air. It is particularly well-suited for thermally unstable compounds like NP, as it does not require high-temperature vaporization. LC-MS/MS is the preferred method for regulatory compliance testing of complex food and biological samples where high sensitivity and specificity are essential. GC-MS is primarily used for volatile and semi-volatile compounds. While it can detect NP after derivatization, it is less suitable for thermally labile or high-molecular-weight compounds. GC-MS excels in the analysis of water and air samples, where derivatization is relatively straightforward, and it remains the standard method for NP detection in many national environmental monitoring programs. HPLC is widely applicable to liquid and solid samples but is most commonly used for water and soil analysis. It is the method of choice for routine screening of large numbers of samples where NP concentrations are above the microgram per liter level. However, HPLC lacks the qualitative capability of mass spectrometry, making it prone to false positives in complex matrices (Table 2).

**Table 2:** Nonylphenol detection methods, applicable media, and advantages and disadvantages.

Detection methods	HPLC	LC-MS/MS	GC-MS	Immunobiotechnology
Typical LOD range	0.08–0.52 μg/L (water) 0.05–0.12 mg/kg (soil)	0.030 μg/kg (water) 0.80 μg/kg (food) 2.7 ng/mL (serum)	0.005 μg/L (water) 0.02 ng/g (cereal) 0.65 μg/kg (oil)	0.12–24.78 μg/L (water)
Sensitivity	Moderate	Excellent	Very good	Low to moderate
Qualitative ability	Poor (Retention time only)	Excellent (MRM mode)	Excellent (Mass spectrum matching)	Poor (Antibody binding only)
Capital cost	Low	Very high	Moderate	Low
Operational cost	Low	High	Moderate	Very low
Analysis time per sample	15–30 min	20–40 min	30–60 min (including derivatization)	5–15 min
Operational complexity	Low	High	High	Very low
Derivatization required	No	No	Yes (for NP)	No
Primary applicable matrices	Water, soil, serum	All matrices (serum, food, water, soil, air)	Water, air, food, sediment	Water, simple food matrices
Main advantages	Widely available, simple operation, low cost	Highest sensitivity, excellent specificity, no derivatization	High separation efficiency, excellent qualitative ability	Ultra-fast, low cost, on-site detection potential
Main disadvantages	Low sensitivity, prone to false positives	High cost, requires trained operators	Requires derivatization, complex operation	High detection limit, risk of cross-reaction

## 4 Detection of nonylphenol in different media

Comparing findings across different media highlights significant variations in method sensitivity and matrix suitability. As summarized in Table 3, while highly sensitive electrochemical immunosensors can achieve ultralow detection limits (e.g., 3.8 ng/L) in liquid leachates from food contact materials, detecting nonylphenol in complex biological or environmental matrices like serum, soil, and air often requires more extensive extraction and purification steps. Methods performing well in relatively straightforward matrices often require additional optimization for complex solid or gaseous matrices to overcome background interference. A comparison of the lowest reported concentrations and detection limits across these media demonstrates the varying analytical requirements for each environment.

**Table 3:** Summary of methods and lowest reported detection limits for nonylphenol across different media.

Medium	Detection method	Lowest reported limit	Reference
Food contact materials	GC-MS	LOD: 0.5 μg/kg	Tang et al. (33)
Food contact materials	Electrochemical immunosensor	LOD: 3.8 ng/L	Wang et al. (34)
Food contact materials	LC-MS/MS	LOQ: 5 μg/kg	Liu et al. (35)
Serum	LC-MS/MS	LOD: 2.7 ng/mL	Kim et al. (36)
Serum	HPLC	LOD: 3.12 ng/mL	Chen et al. (37)
Soil/Sediment	HPLC	Detected: 0.0523 mg/kg	Wang et al. (38)
Soil/Sediment	GC-MS/MS	LOD: 0.08–1.5 μg/kg	Novotná et al. (39)
Air	GC-MS/MS	LOD: ~0.5 pg	Li et al. (40)
Air	LC-MS/MS	Detected: 0.213 ng/m3	Zhang et al. (42)

LOD, Limit of Detection; LOQ, Limit of Quantification.

### 4.1 Detection of nonylphenol in food contact materials

Nonylphenol polyoxyethylene ether is often used as a plastic additive in food contact materials, and food packaging bags are inseparable from daily human life. When nonylphenol polyoxyethylene ether decomposes, the residual nonylphenol in food contact materials can enter the human body through the digestive tract, jeopardizing human health. Hence, detecting nonylphenol in food packaging is particularly important. Tang et al. (35) used gas chromatography-mass spectrometry (GC-MS) to detect nonylphenol in plastic bags, determining a method detection limit of 0.5 μg/kg and a quantification limit of 1.0 μg/kg. The average recoveries at three spiked levels (1.0, 10.0, and 200.0 μg/kg) ranged from 89.2% to 101.2%, with relative standard deviations (RSD) of 3.1% to 6.2%. These results show that the method is reliable, sensitive, and suitable for determining nonylphenol in food bags. Furthermore, an ultrasensitive electrochemical immunosensor for detecting nonylphenol in leachate from food contact materials was developed by Wang et al. (36). They self-assembled gold nanoclusters with Ti3C2 MXene to modify a glassy carbon electrode (GCE), which exhibited excellent conductivity. Anti-nonylphenol antibodies were immobilized on the modified electrode; upon specific binding to nonylphenol, the conductivity of the GCE significantly reduced. This immunosensor is characterized by a remarkably low detection limit (3.8 ng/L) and high specificity. Additionally, Liu et al. (37) used UPLC-MS/MS to determine nonylphenol in 17 common food contact materials, such as plastic wraps, bread bags, and condiment bags, achieving a lower limit of quantification of 5 μg/kg. The average spiked recoveries at levels of 10, 100, and 200 μg/kg ranged from 86.3% to 90.5% with an RSD of <12%, indicating that the LC-MS/MS technique offers high sensitivity and is highly suitable for this application.

### 4.2 Detection of nonylphenol in serum

Nonylphenol has toxic effects on organisms; therefore, directly detecting its levels in biological systems is essential for preventing and treating nonylphenol-induced diseases. Detecting nonylphenol in animal serum is also a necessary means of studying its dose-effect relationship. Kim et al. (38) used a novel automated hybrid solid-phase extraction-precipitation technique (hybrid SPE-PPT) to extract and purify nonylphenol from human serum. They first centrifuged the samples and added protein precipitants, and the supernatant was then directly processed using a hybrid SPE column, which allowed the extraction step to be completed in less than 40 s, avoiding time-consuming traditional SPE steps. The supernatant was analyzed by LC-MS/MS with a limit of detection (LOD) of 2.7 ng/mL, making it highly suitable for high-throughput serum analysis due to its sensitivity and time-saving features. Similarly, Chen et al. (39) successfully applied HPLC to determine nonylphenol in rat serum, using n-hexane and ethyl acetate as extractants. The LOD was 3.12 ng/mL, with recoveries ranging from 86.2% to 99.7%. The intra-day precision was 0.28%−1.15%, and the inter-day precision was 0.87%−1.76% (both <2%). In conclusion, the HPLC technique is easy to operate, reliable, sensitive, yields high recovery rates, and is well-suited for determining nonylphenol in animal serum.

### 4.3 Detection of nonylphenol in soil

Nonylphenol in soil is primarily derived from the degradation of various pesticides. In addition, most nonylphenol in wastewater treatment plants attaches to sludge and enters the environment through sludge disposal routes. Monitoring the soil environment can thus help mitigate hazards to human health. Wang et al. (40) used HPLC to detect nonylphenol in agricultural soil long-term sprayed with biopesticides, finding NP concentrations ranging from 0.0523 mg/kg to 0.1247 mg/kg. Average recoveries ranged from 80.2% to 82.7%, with RSDs between 4.8% and 5.52%, demonstrating that HPLC is highly suitable for detecting nonylphenol in pesticide-treated soils. Furthermore, Novotná et al. (41) used GC-MS/MS to determine nonylphenol in river and lake sediments. The method achieved recoveries of 75%−98%, a detection limit of 0.08–1.5 μg/kg, and a quantification limit of 0.3–4.0 μg/kg, proving that GC-MS/MS analysis for solid sediments can be fast, simple, and cost-effective.

### 4.4 Detection of nonylphenol in air

Currently, research on the detection of nonylphenol in air has been gradually carried out in China, with in-depth studies also conducted in Europe and North America. Li et al. (42) utilized GC-MS/MS for the analysis of atmospheric particulate matter, reporting an LOD in the range of 0.5–2 pg, precision between 2% and 15%, and recoveries ranging from 62% to 85%. Their study detected NP in 92% of urban atmospheric particulate samples from 12 cities in China, revealing the widespread occurrence of NP in the atmospheric environment. González-Gálvez et al. (43) examined airborne particulate samples collected in urban and industrial areas of Spain, revealing that nonylphenol was present in all samples, with concentrations ranging from 0.02 to 1.37 ng/m3. Additionally, Zhang et al. (44) analyzed atmospheric precipitation and particulate matter samples from North China using LC-MS/MS, successfully detecting nonylphenol at a mean concentration of 0.213 ng/m3 in particulate matter, and revealed significant seasonal variation characteristics.

## 5 Bioremediation of nonylphenol

While robust pretreatment and sensitive detection methods are critical for identifying nonylphenol contamination, their ultimate practical application lies in monitoring and validating environmental remediation efforts. Nonylphenol accumulates in the environment and poses long-term health risks if not degraded. Consequently, bioremediation—utilizing organisms such as fungi, bacteria, and plants to remove nonylphenol—has emerged as a promising approach. However, evaluating the success of these biological interventions inherently relies on the analytical techniques discussed earlier in this review. For instance, determining precise degradation kinetics requires highly accurate monitoring. Lara-Moreno et al. (45) isolated specific degrading bacteria from sewage sludge using cyclodextrins as availability enhancers. Relying on accurate detection methods, they determined that Bacillus subtilis CN12 was highly effective, with a DT50 (time to half the initial concentration) of only 0.9 days and complete degradation in less than seven days. Similarly, verifying detoxification in complex matrices like sediments demands rigorous sample pretreatment. Zhang et al. (46) demonstrated the bioremediation of nonylphenol-contaminated sediments via composting with *Phanerochaete chrysosporium* inoculums. By optimizing the C/N ratio, they confirmed that the sediments were effectively detoxified after 90 days, with a degradation rate of over 92%. In another study, Hung et al. (47) evaluated cyanobacterial biochar as a carbon-neutral material to enhance NP removal from waste-activated sludge, tracking a 75% removal of nonylphenol when utilizing a specific dose (1.7 g/L, pH 6, 500 °C). These results suggest that while bioremediation can significantly degrade nonylphenol, removal efficiency varies widely depending on the biological agent and the complexity of the environmental matrix. Therefore, the rigorous sample pretreatment and highly sensitive detection methods detailed in earlier sections are indispensable. Without them, it would be impossible to accurately track degradation kinetics, optimize activator dosing, or verify the complete removal of nonylphenol during future bioremediation research.

## 6 Conclusion

Accurate monitoring of nonylphenol across diverse environmental matrices requires tailored analytical strategies, as no single technique is universally applicable. This review highlights key comparative findings in sample pretreatment and detection. For pretreatment, solid-phase extraction (SPE) and liquid-liquid extraction (LLE) are highly effective for aqueous samples despite potential clogging or emulsion issues, whereas Soxhlet extraction provides robust recovery for solids but remains time- and solvent-intensive. Solid-phase microextraction (SPME) offers a versatile, solvent-free alternative across solid, liquid, and gaseous media, though fiber fragility limits its durability. Regarding practical implications for method selection, chromatographic methods (HPLC, LC-MS, and GC-MS) remain the gold standard for complex matrices (e.g., soil, serum, air, and food) due to their exceptional sensitivity and nanogram-level detection limits. However, their practical application is often constrained by high equipment costs, cumbersome sample preparation, and lengthy processing times. Conversely, immunobiological techniques (e.g., ELISA) provide a highly specific, rapid, and cost-effective alternative for simpler matrices like water. While they currently exhibit higher detection limits than instrumental methods, their ease of use makes them an ideal screening tool. Moving forward, future research must address several critical knowledge gaps. First, efforts should focus on overcoming the inherent limitations of current methods by accelerating the processing speeds and reducing the costs of instrumental analyses, while simultaneously enhancing the sensitivity of immunobiological assays. Second, while this review establishes a framework for method selection, the specific impact of different adsorption column types on extraction efficiency was not fully explored. Investigating these variables in future studies will be essential for optimizing extraction protocols, improving recovery rates, and advancing nonylphenol detection toward greater precision, efficiency, and simplicity (Table 4).

**Table 4:** Bioremediation of nonylphenol.

Bioremediation of nonylphenol	Degradation condition	Removal effects
*Bacillus subtilis* CN12	Cyclodextrins as enhancers	DT50 degradation value is 0.9 days and complete degradation time is less than 7 days
*Phanerochaete chrysosporium* inoculums	Inoculum composting with optimized C/N ratio (25:1)	Effective detoxification of nonylphenol-contaminated sediments after 90 days of composting, degradation rate over 92%
Cyanobacterial biochar coupled with sodium sulfite	500 °C prepared biochar, dosed at 1.7 g/L, pH 6	Removal of 75% of nonylphenol from waste-activated sludge
